# P-1530. Microbiome factors associated with fluoroquinolone-resistant Enterobacterales bloodstream infection in hematopoietic cell transplant recipients receiving fluoroquinolone prophylaxis

**DOI:** 10.1093/ofid/ofaf695.1711

**Published:** 2026-01-11

**Authors:** Grace A Maldarelli, Jamie Marino, John Lee, Lars Westblade, Michael Hovan, Claire Douglass, Emily Davidson, Randy S Longman, Michael J Satlin

**Affiliations:** Weill Cornell Medicine, New York, NY; Weill Cornell Medicine, New York, NY; Weill Cornell Medicine, New York, NY; Weill Cornell Medicine, New York, NY; NewYork-Presbyterian Hospital/Weill Cornell Medical Center, New York City, New York; NewYork-Presbyterian Hospital/Weill Cornell Medical Center, New York City, New York; University of Maryland Medical Center, Baltimore, Maryland; Jill Roberts Center for IBD, Division of Gastroenterology and Hepatology, Department of Medicine, Weill Cornell Medicine, New York, New York; Weill Cornell Medicine, New York, NY

## Abstract

**Background:**

Hematopoietic cell transplantation (HCT) is potentially curative for hematologic malignancies, but leads to a profound neutropenia that predisposes to bloodstream infections (BSIs) due to Enterobacterales. While fluoroquinolone (FQ) prophylaxis decreases risk of Enterobacterales BSI, HCT recipients colonized with fluoroquinolone-resistant Enterobacterales (FQRE) prior to HCT frequently develop Enterobacterales BSI despite FQ prophylaxis. We hypothesized that there are differences in microbiome composition between FQRE-colonized and non-colonized patients, and between colonized patients who develop FQRE BSI and those who do not.

**Figure:**
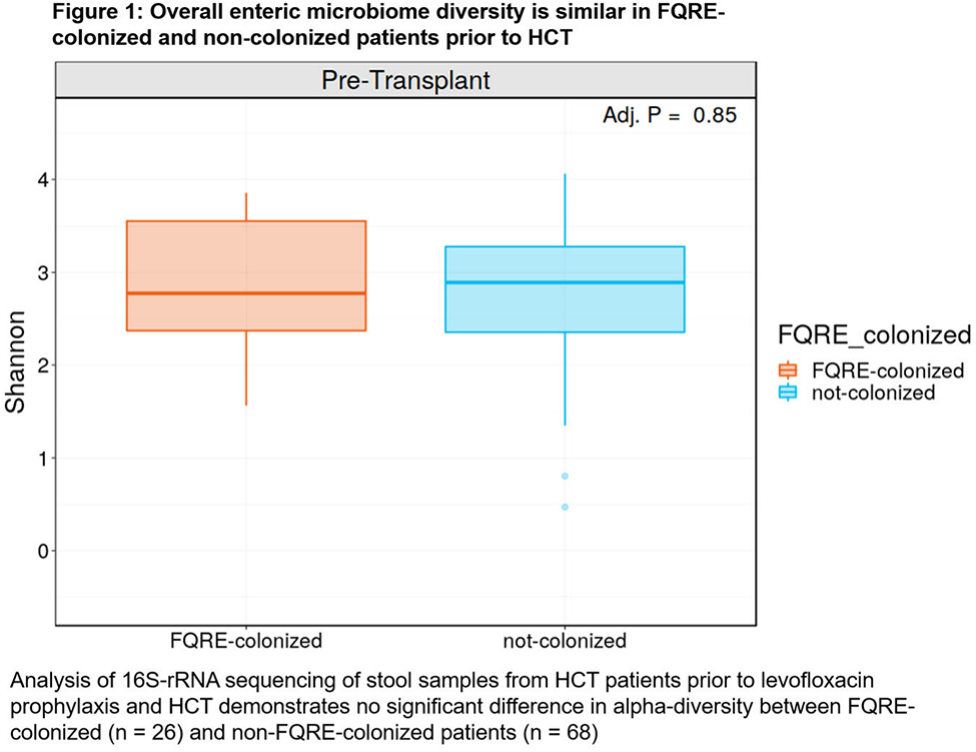

**Figure:**
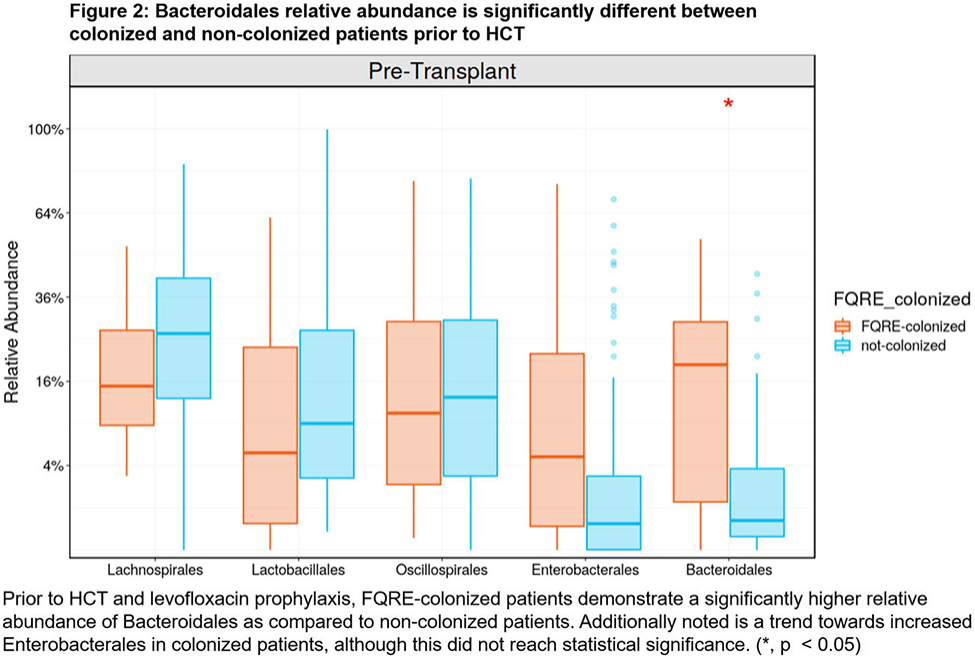

**Methods:**

We collected stool specimens during pre- and post-HCT weeks from patients undergoing HCT from 2016-2019 at a single center. Specimens underwent quantitative culturing for FQRE. DNA was isolated for 16S rRNA sequencing. We evaluated microbiome diversity and composition between FQRE-colonized and non-colonized patients, and between colonized patients who did or did not develop FQRE BSI.

**Figure:**
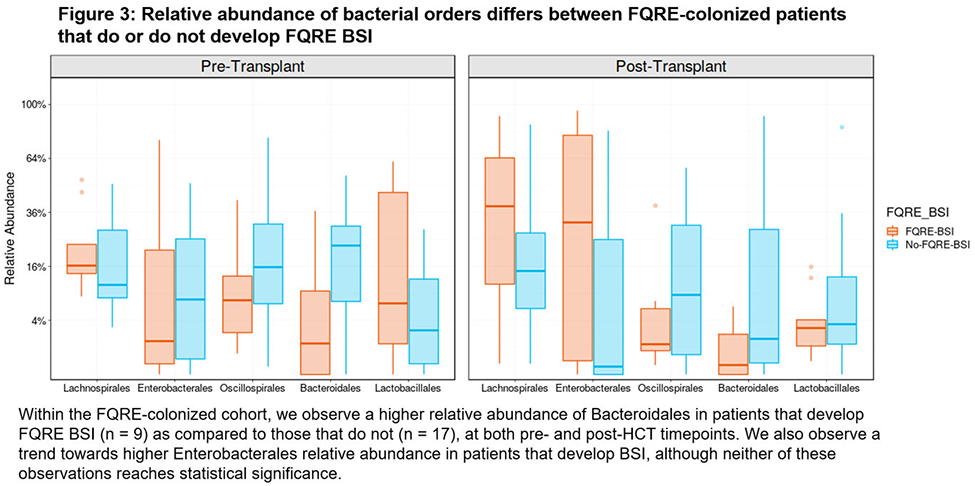

**Results:**

Stool specimens were collected from 26 FQRE-colonized patients and 68 non-colonized patients. FQRE-colonized patients had similar alpha-diversity to non-colonized patients (Fig. 1), but showed higher relative abundance of Bacteroidales prior to HCT (Fig. 2, p < 0.05). In FQRE-colonized patients, alpha-diversity, Enterobacterales relative abundance, and FQRE colonization density were similar pre-HCT between patients who did or did not develop FQRE BSI. Post-HCT, we observe a higher relative abundance of Enterobacterales in patients who developed FQRE BSI compared to those who did not. Additionally, patients who developed FQRE BSI had lower relative abundance of Bacteroidales both before and after HCT (Fig. 3). However, neither observation reaches statistical significance.

**Conclusion:**

FQRE-colonized patients demonstrate higher relative abundance of Bacteroidales than non-colonized patients prior to HCT; increased relative abundance of Bacteroidales was protective against FQRE BSI for FQRE-colonized patients. Further work is needed to validate these findings and investigate the mechanism by which Bacteroidales may decrease the risk of FQRE BSI in colonized patients.

**Disclosures:**

John Lee, MD, Astellas: Honoraria|BioFire Diagnostics, LLC: Grant/Research Support|Calliditas: Travel support|Eurofins Viracor: Patent US-2020-0048713-A1 licensed to Eurofins Viracor Lars Westblade, PhD, Elements Materials Technology: Grant/Research Support|Hardy Diagnostics: Grant/Research Support|Melinta Therapeutics: Grant/Research Support|Selux Diagnostics: Grant/Research Support|Shionogi: Advisor/Consultant|SNIPRBIOME: Grant/Research Support Randy S. Longman, MD, PhD, Boeringer Ingelheim: Grant/Research Support|CJ Biosciences: Advisor/Consultant|Pfizer: Advisor/Consultant|Sanofi: Advisor/Consultant|Xencor: Advisor/Consultant Michael J. Satlin, MD, MS, AbbVie: DSMB participant|bioMerieux: Grant/Research Support|Merck: Grant/Research Support|SNIPRBiome: Grant/Research Support

